# Planned en bloc resection using veno‐venous bypass for an extensive retroperitoneal sarcoma with a caval thrombus in a dog

**DOI:** 10.1111/vsu.70026

**Published:** 2025-09-18

**Authors:** Tatsuya Heishima, Kumiko Ishigaki, Takeo Ueda, Yumiko Kagawa, Kaito Iida, Ryo Takeuchi, Kazushi Asano

**Affiliations:** ^1^ Laboratory of Veterinary Surgery, Department of Veterinary Medicine, College of Bioresource Sciences Nihon University Fujisawa Kanagawa Japan; ^2^ Upfield Veterinary Hospital Kyoto Kyoto Japan; ^3^ North Lab Sapporo Hokkaido Japan

## Abstract

**Objective:**

To report the successful complete resection of an extensive retroperitoneal sarcoma (RPS) with a caval thrombus using veno‐venous bypass (VVB) to facilitate temporary caudal vena cava (CVC) occlusion.

**Study design:**

Case report.

**Animal:**

A 12‐year‐old neutered male Standard Poodle.

**Methods:**

Computed tomography (CT) revealed an extensive mass originating from the right adrenal gland region with an associated caval thrombus extending into the thoracic CVC. A preemptively planned en bloc resection, including the right kidney and caval thrombus, was performed. VVB and a Pringle maneuver were used to minimize hemorrhage and maintain hemodynamic stability.

**Results:**

Surgery was successfully completed without major complications. The operative time was 161 min, with Pringle maneuver duration of 4 min 8 s and a caval occlusion time of 44 min 43 s. The dog's general condition stabilized the following day with no decline in renal function. The histopathologic examination confirmed the diagnosis of RPS. The dog remained in good health with no recurrence or metastasis at the 1‐year follow‐up.

**Conclusion:**

This is the first report of a clinical application of VVB during temporary caval occlusion to achieve complete en bloc resection of an extensive adrenal RPS with ipsilateral kidney and caval thrombus, followed by repair of a CVC incision. VVB may be a valuable technique for maintaining hemodynamic stability and reducing hemorrhage during complex oncological surgeries requiring temporary caval occlusion.

## 1 INTRODUCTION

Tumor thrombus formation into the caudal vena cava (CVC) is frequently associated with canine adrenal tumors, particularly pheochromocytoma (PHEO), occurring in approximately 10%–40% cases.^1^, ^2^, ^3^, ^4^, ^5^, ^6^ However, although rare, tumor thrombus formation has also been reported in dogs with retroperitoneal sarcoma (RPS).^7^ When removing extensive caval thrombus, it is necessary to use a temporary occlusion of venous blood flow for the reconstruction of CVC. In humans, this can cause acute renal failure^8^ and intraoperative hypotension due to decreased venous return.^9^

The use of veno‐venous bypass (VVB) has been shown to stabilize patient hemodynamics during the temporary occlusion of the inferior vena cava in human medicine.^3^ Previous reports have described the use of VVB during surgery, including total pancreatectomy^10^ and liver transplantation.^11^ For creating VVB, an Anthron bypass tube (Toray Industries, Inc., Tokyo, Japan) can be applied.^12^, ^13^ It consists of a heparinized hydrophilic tube that releases small amounts of heparin into the bloodstream as it flows through the tube, providing anticoagulant effects within the tube while having minimal impact on the body's overall coagulation status. In veterinary medicine, there have been no reports on the use of VVB in clinical settings. However, VVB has been used in experimental dogs to study human liver transplantation.^14^, ^15^

Therefore, this case report aimed to report a successful complete resection of an extensive RPS with a caval thrombus using VVB for the temporary occlusion of CVC.

## 2 MATERIALS AND METHODS

**FIGURE 1:**
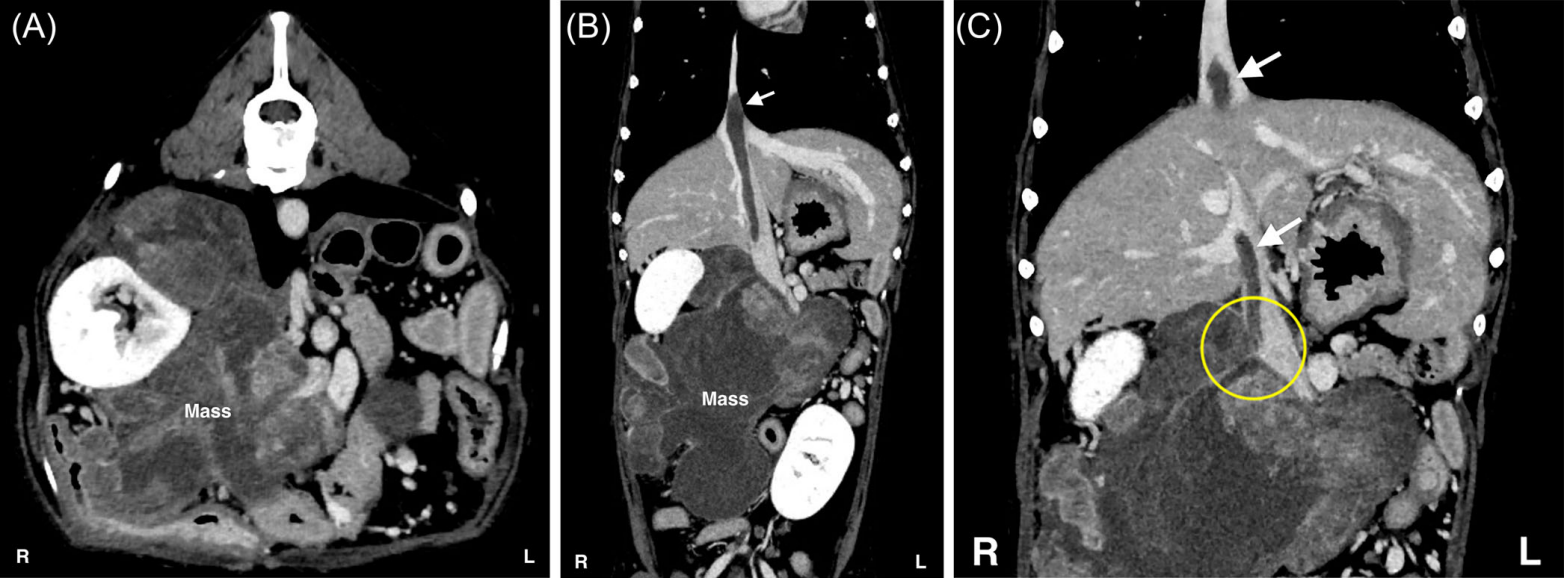
Preoperative computed tomography (CT) findings. (A) Axial image. (B) Coronal image. (C) Coronal image highlighting the origin of the caval thrombus. An extensive mass was observed in the right adrenal region, compressing the caudal vena cava (CVC) from the caudal aspect of the liver and involving the right kidney, right renal artery, and vein. The right adrenal gland was not identifiable. The tumor exhibited low contrast enhancement on CT angiography, with no significant changes noted on triple‐phase contrast‐enhanced CT. The caval thrombus (white arrow) originated from the right phrenicoabdominal vein (yellow circle) and extended cranially beyond the diaphragm into the intrathoracic CVC.

A 12‐year‐old castrated male Standard Poodle was referred with a 2‐month history of anorexia, lethargy, and abdominal distention. The dog weighed 17.7 kg and had a body condition score of 2 out of 5. Blood tests (Table 1) showed normal renal function and no evidence of anemia. Urinalysis revealed proteinuria (30 mg/dL) with a specific gravity of 1.028, and no other abnormalities were noted. Radiography and ultrasonography revealed a large mass in the cranial abdomen. Computed tomography (CT) revealed an extensive mass of approximately 15 cm in diameter, compressing the CVC from the caudal aspect of the liver and affecting the right kidney, right renal artery, and vein (Figure 1). The right adrenal gland was not recognized. The inside of the tumor showed low‐contrast enhancement on CT angiography, with no marked changes on triple‐phase contrast‐enhanced CT. Furthermore, a caval thrombus was detected extending from the vicinity of the right phrenicoabdominal vein to beyond the diaphragm to the thoracic CVC. An adrenocorticotropic hormone (ACTH) stimulation test revealed blood cortisol levels of 2.3 and 14.8 μg/dL before and after stimulation, respectively (reference range: 1.0–6.0 μg/dL). The plasma catecholamine fractions were as follows: adrenaline, 73 pg/mL (reference range: < 100 pg/mL); noradrenaline, 573 pg/mL (reference range: < 450 pg/mL); and dopamine, 71 pg/mL (reference range: < 20 pg/mL).

**TABLE 1:** Blood test findings.

Variables	Unit	Initial examination	POD 1	POD 13	POD 20	Normal range
Complete blood count						
PCV	%	38	39	36	39	37–55
Hb	g/dL	12.9	13.3	12.4	12.7	12.0–18.0
RBC	10^6^/mL	5.60	547	509	5.46	5.50–8.50
WBC	/mL	9200	29 400	6900	8100	6000–17 000
Plt	10^3^/mL	506	313	404	293	200–400
Serum chemistry						
TP	g/dL	6.7	4.7	6.1	6.1	5.2–8.2
Alb	g/dL	3.4	2.6	3.5	3.6	2.7–3.8
AST	U/L	40	223	27	45	0–50
ALT	U/L	55	36	50	105	10–100
ALP	U/L	41	115	74	74	23–212
GGT	U/L	4	4	13	10	0–7
Amy	U/L	1010	697	915	761	200–1400
Lipa	U/L	78	76	385	83	10–160
BUN	mg/dL	16.1	8.7	24.4	51.0	7.0–21.0
Cr	mg/dL	0.66	0.97	1.02	1.03	0.5–1.8
SDMA	μg/dl	11	NA	18	NA	0–14
TCho	mg/dl	246	139	254	260	115–337
Glu	mg/dL	91	136	99	93	77–125
Na	mmol/L	147	145	148	150	134–153
K	mmol/L	3.8	4.5	3.8	4.5	3.4–4.6
Cl	mmol/L	113	113	111	116	105–118
Ca	U/L	10.2	9.6	11.1	10.3	9.3–12.1
P	U/L	3.5	5.7	4.2	4.3	1.9–5.0
CRP	mg/dL	3.05	NA	0.1	0.05	0–1.00
Coagulation test						
APTT	s	11.8	16.3	NA	NA	10.0–16.0
PT	s	5.2	6.4	NA	NA	6.0–8.0
Fib	mg/dL	387.4	280.7	NA	NA	86.0–375.0
AT	%	127	81	NA	NA	102–156
D‐dimer	mg/dL	1.93	7.05	NA	NA	0–2.00

Abbreviations: Alb, albumin; ALP, alkaline phosphatase; ALT, alanine aminotransferase; Amy, amylase; APTT, activated partial thromboplastin time; AST, aspartate aminotransferase; AT, anti‐thrombin activity; BUN, blood urea nitrogen; Cr, creatinine; CRP, C‐reactive protein; Fib, fibrinogen; GGT, gamma‐glutamyl transferase; Glu, glucose; Hb, hemoglobin; Hb, hemoglobin; Lipa, lipase; PCV, packed cell volume; Plt, platelet count; POD, postoperative day; PT, prothrombin time; RBC, red blood cell count; SDMA, symmetric dimethylarginine; TCho, total cholesterol; TP, total protein; WBC, white blood cell count.

Based on the findings of the CT images, a pheochromocytoma with a caval thrombus was suspected based on the anatomical location of the extensive mass. Since the tumor was anticipated to be invading or adherent to the surrounding tissues, the tumor dissection from the surrounding tissues was expected to prolong the time required for the CVC occlusion. Additionally, as the right kidney had to be removed, preserving the function of the left kidney was of critical importance. Therefore, VVB was planned to allow safe surgical manipulation during the CVC occlusion. Six days after the first visit, complete resection of the abdominal mass was performed after obtaining the owner's consent.

The anesthesia protocol included subcutaneous administration of 1.0 mg/kg prednisolone (Kyoritsu Pharmaceutical Co., Ltd., Tokyo, Japan), 1.0 mg/kg maropitant citrate hydrate (Zoetis Japan Co., Ltd., Tokyo, Japan) and 5.0 μg/kg fentanyl hydrochloride (Terumo Co., Tokyo, Japan). General anesthesia was induced by intravenous propofol (Nichi‐Iko Co., Ltd., Toyama, Japan), and maintained with sevoflurane (Zoetis Japan Co., Ltd.). Intraoperative continuous rate infusion (CRI) was as follows: 2.5–5.0 μg/kg/min of dopamine hydrochloride, 2.5–5.0 μg/kg/min of dobutamine hydrochloride (Kyowa Pharmaceutical Co., Ltd., Osaka, Japan), and 0.05 μg/kg/min of carperitide (Daiichi Sankyo Co., Ltd., Tokyo, Japan) for intraoperative circulatory support; 10–40 μg/kg/h of remifentanil hydrochloride (Maruishi Pharmaceutical Co., Ltd., Osaka, Japan) and 50 μg/kg/min lidocaine (Sand Farm Co., Ltd., Tokyo, Japan) for intraoperative analgesia. In addition, nafamostat mesylate (Nichi‐Iko Co., Ltd.) was continuously administered at 0.2 mg/kg/h throughout the surgery. To manage intraoperative hypotension, CRI of phenylephrine (Kowa Co., Ltd., Tokyo, Japan) and noradrenaline (Alfresser Pharma Co., Ltd., Osaka, Japan) were performed as needed.

**FIGURE 2:**
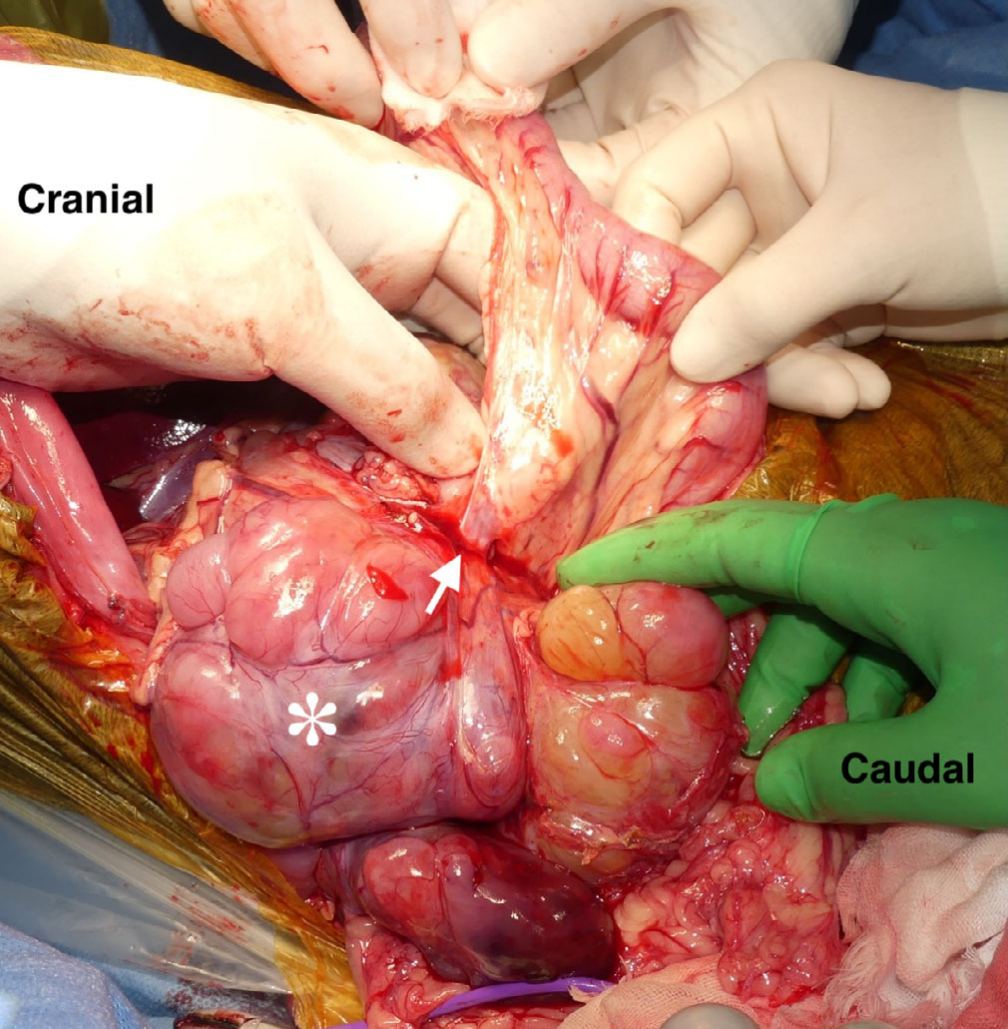
Intraoperative findings. The extensive mass (*) was found to be adherent to the duodenal ligament (white arrow) and the right lobe of the pancreas.

**FIGURE 3:**
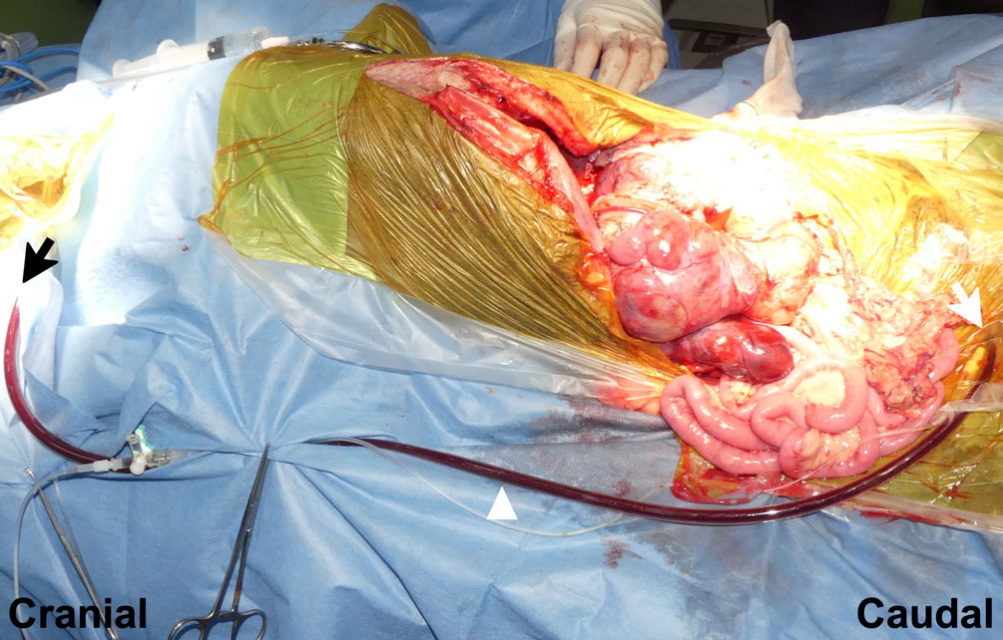
Veno‐venous bypass between the right jugular and femoral veins. An Anthron bypass tube (VVT‐4860; Toray Industries Inc.; arrowhead) filled with saline was inserted into the right femoral vein (white arrow), and the other saline‐filled bypass tube was similarly placed into the right jugular vein (black arrow). The two tubes were then connected to form a single conduit, creating a temporary bypass of the caudal vena cava (CVC) from the femoral to the jugular vein.

**FIGURE 4:**
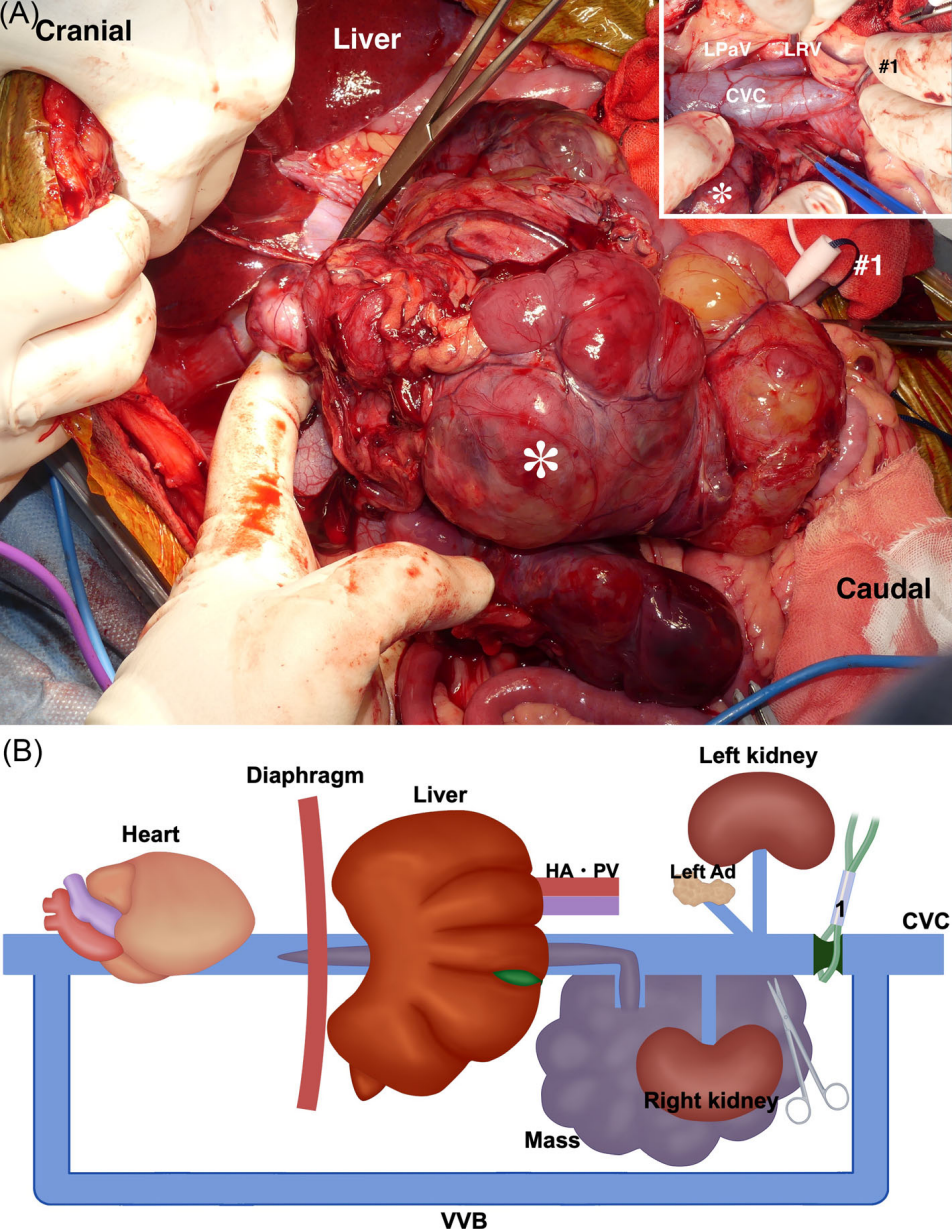
Surgical dissection of the caudal side of the mass from the caudal vena cava (CVC). (A) Intraoperative findings. (B) Schematic illustration. The image in the upper right of (A) shows the dissection of the mass (*) from the CVC during the tourniquet (#1) was tightened. After the tourniquet (#1) was tightened, the whole mass (*) was grossly inspected and carefully dissected from the CVC up to the cranial side of the left renal vein. Ad, adrenal gland; HA, hepatic artery; LRV, left renal vein; LPaV, left phrenicoabdominal vein; PV, portal vein; VVB, veno‐venous bypass.

**FIGURE 5:**
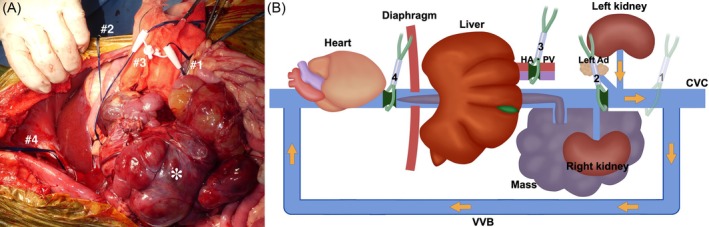
The sites where the tourniquets (#1–4) were applied for the dissection of the mass from the caudal vena cava (CVC). (A) Intraoperative findings. (B) Schematic illustration. Blood flow was sequentially occluded using tourniquets in the following order: The CVC cranial to the left renal and phrenicoabdominal veins (#2), Pringle maneuver (#3), and the thoracic CVC (#4). Subsequently, the tourniquet (#1) on CVC caudal to the mass (*) was released. During these occlusions, venous drainage from the left kidney was maintained via veno‐venous bypass (VVB), as indicated by the yellow arrow in (B). The CVC near the right phrenicoabdominal vein was then incised for the removal of caval thrombus. Ad, adrenal gland; HA, hepatic artery; PV, portal vein.

**FIGURE 6:**
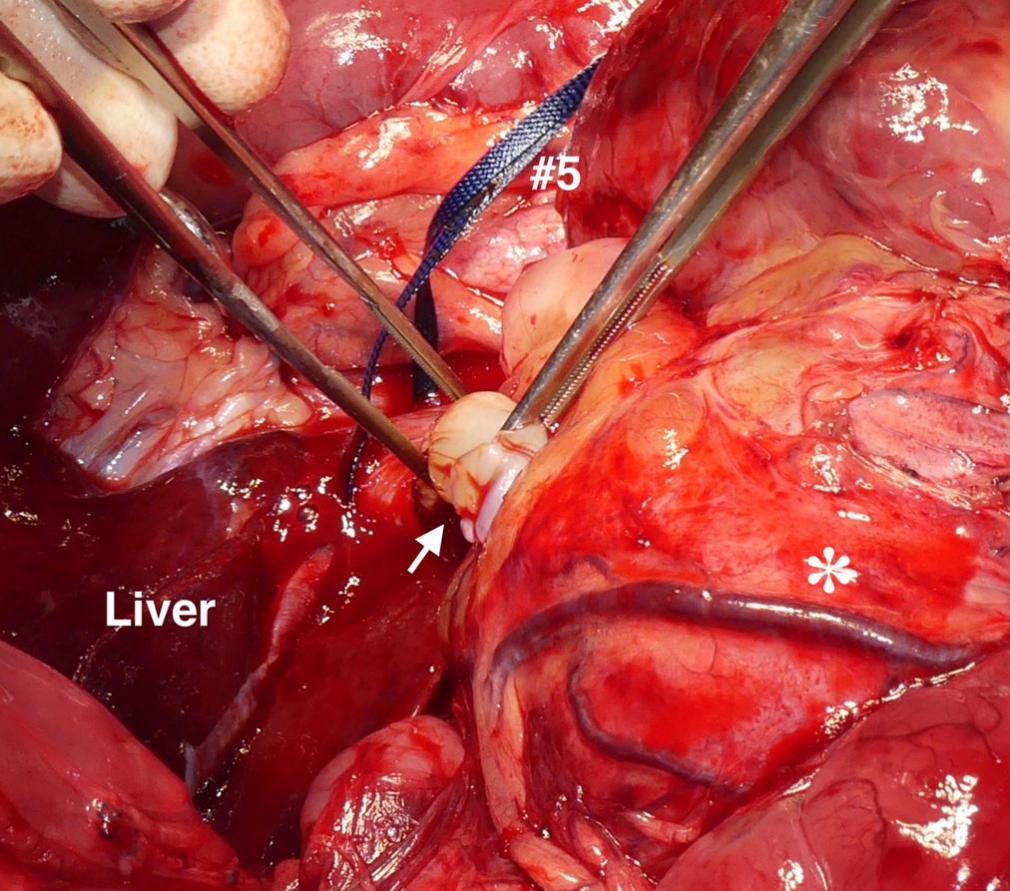
Surgical removal of the caval thrombus. Following the caval incision near the right phrenicoabdominal vein, a new tourniquet (#5) was placed on the caudal vena cava (CVC) between the liver and the site of caval incision. Then, the caval thrombus (white arrow) was removed from the caval incision.

The dog was positioned in dorsal recumbency under general anesthesia. A Mercedes incision (a combination of cranial midline celiotomy and bilateral paracostal incisions) was performed to approach the mass. To expose the entirety of the mass, a caudal median sternotomy and a diaphragmatic midline incision were added. The mass was found to be adherent to the duodenal ligament and the right lobe of the pancreas (Figure 2). It was also firmly attached from the caudal aspect of the liver to the caudal side of the right kidney along the CVC, with extensive adhesion. A small skin incision was made in the right femoral triangle to isolate the femoral artery and vein. A 6‐Fr catheter (Atom multipurpose tube; Atom Medical Co., Tokyo, Japan) was placed in the femoral artery for intraoperative arterial pressure monitoring. An additional small incision was made in the cervical region to isolate the right jugular vein. An Anthron bypass tube (VVT‐4860; Toray Industries Inc.) prefilled with saline was inserted into the right femoral vein, and the other saline‐filled bypass tube was similarly placed into the right jugular vein. The two tubes were then connected to form a single conduit, creating a temporary bypass of the CVC from the femoral to the jugular vein (Figure 3). The Anthron bypass tube used in this case was a tapered tube, measuring 60 cm in length, with an outer diameter of 4 mm at both ends. A tourniquet (#1) was applied to the CVC caudal to the mass, and following its occlusion, the mass was carefully dissected from the CVC up to the level of the left renal vein (Figure 4). In addition, the right ureter was ligated and transected. Three tourniquets were subsequently applied in the following order: (#2) on the CVC cranial to the left renal vein, (#3) around the Glisson's capsule at the hepatic hilum for the Pringle maneuver to occlude hepatic arterial and portal venous inflow, and (#4) on the intrathoracic CVC cranial to the thrombus (Figure 5). The initially placed tourniquet (#1) was then released, and the three tourniquets were tightened sequentially in the order of: cranial to the left renal vein, Pringle maneuver, and cranial to the caval thrombus. These vascular occlusions combined with VVB were implemented not only to minimize hemorrhage during thrombus removal but also to preserve the venous return from the left kidney via the VVB to the right jugular vein. While the CVC was clamped, an incision was made in the CVC near the right phrenicoabdominal vein. A new tourniquet (#5) was placed on the CVC between the liver and the site of caval incision, and the caval thrombus was removed from the caval incision (Figure 6). After the thrombus was removed from the CVC, the new tourniquet (#5) was tightened. The tourniquets for the Pringle maneuver (#3) and on the intrathoracic CVC (#4) were both released. The mass was dissected from the surrounding tissues including the CVC after the right renal artery, vein, and ureter were ligated and transected and the right phrenicoabdominal vein was also resected. The mass was completely removed en bloc with the right kidney and thrombus.

**FIGURE 7:**
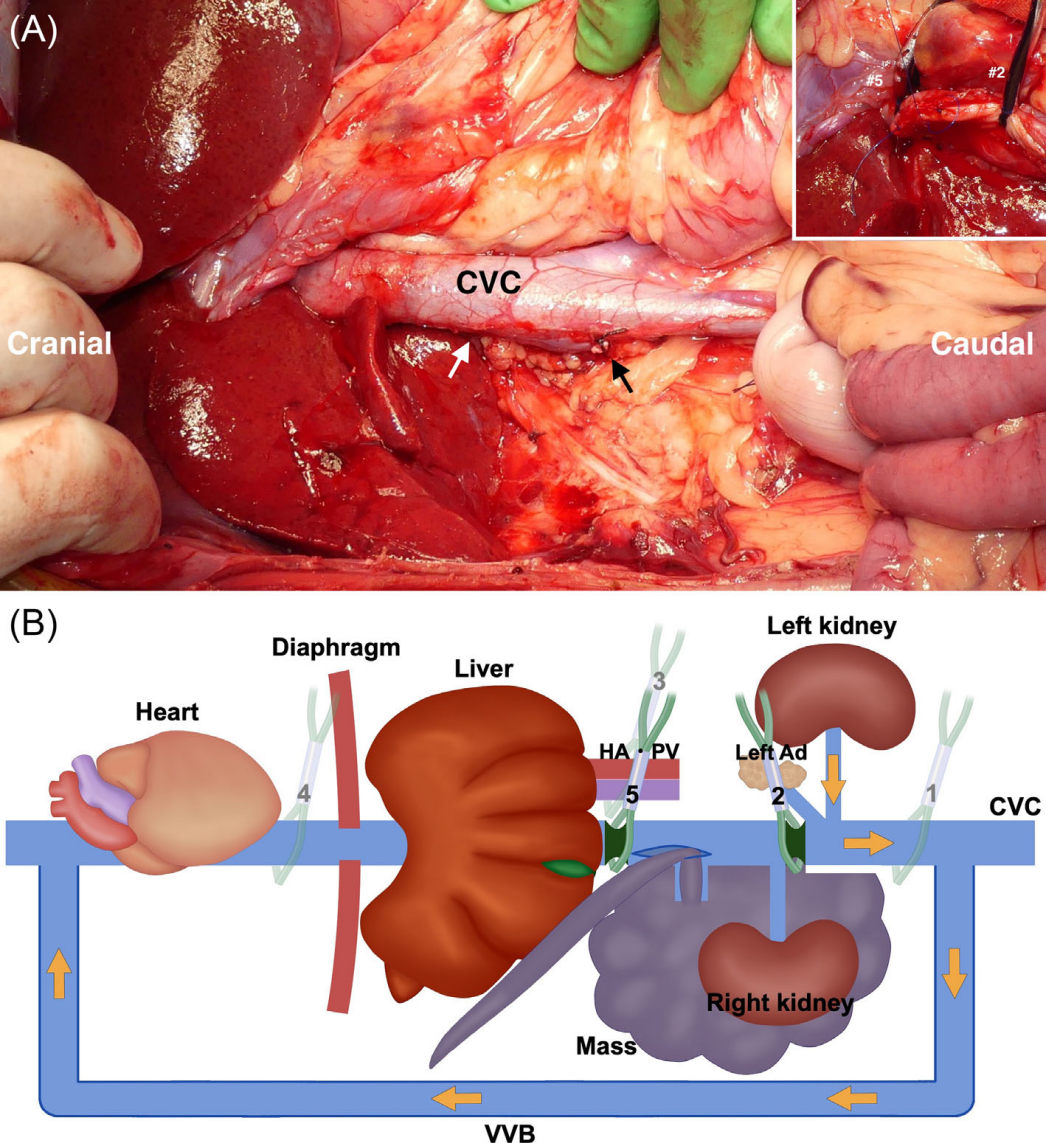
Surgical removal of the tumor with the caval thrombus and reconstruction of the caudal vena cava (CVC). (A) Intraoperative findings. (B) Schematic illustration. Immediately after removal of the thrombus from CVC, the post‐hepatic CVC is occluded using a tourniquet (#5), as illustrated in (B). Subsequently, the tourniquets (#3 and #4) are released, allowing portal venous and hepatic arterial blood to perfuse the liver and drain into CVC via the hepatic veins. The right renal vein (black arrow) was ligated and transected, enabling en bloc resection of the tumor involving the right kidney. The incision site of CVC (white arrow) was then reconstructed using a continuous suture pattern with 5–0 polypropylene. The image in the upper right of (A) shows the reconstruction with the continuous suture pattern. After reconstruction, the remaining tourniquets (#2 and #5) are released, and no bleeding is observed. Ad, adrenal gland; HA, hepatic artery; PV, portal vein; VVB, veno‐venous bypass.

The CVC was reconstructed using a continuous suture pattern with 5–0 polypropylene (PROLENE Hemo‐Seal, Johnson & Johnson, New Brunswick, New Jersey). Following this, the tourniquets cranial (#5) and caudal (#2) to the mass were released, and normal blood flow through the CVC was restored without bleeding (Figure 7). To terminate the VVB, the connected bypass tube was first withdrawn from the right femoral vein, and both proximal and distal ends of the venotomy were ligated to achieve hemostasis. The tube was then elevated to allow residual blood to drain into the right jugular vein by gravity, after which it was removed from the jugular vein and the venotomy was similarly ligated. Finally, the diaphragm was reconstructed, a thoracostomy tube was placed, and the caudal median sternotomy was routinely closed, followed by routine closure of the abdominal incision. For additional postoperative analgesics, ultrasound‐guided paravertebral blocks were performed at six sites, bilaterally at the levels of the ninth, 11th, and 13th thoracic vertebrae, with bupivacaine administered at a dose of 0.25 mg/kg per site.

## 3 RESULTS

No major complications were observed during the surgery, and the operative time from the start of the skin incision to the end of the abdominal closure was 161 min. During the operation, the time required for the Pringle maneuver was 4 min 8 s, and the clamp time for CVC was 44 min 43 s. The total perioperative blood transfusion volume was 200 mL.

No significant change was observed in the mean arterial blood pressure on the caudal side of the left renal vein before and after CVC occlusion (57 vs. 58 mmHg). The mean blood pressure dropped to 33 mmHg during the Pringle maneuver for 4 min and 8 s. However, it increased to 51 mmHg after releasing the Pringle maneuver. To remove the extensive mass, CVC occlusion on the caudal side of the liver and the cranial side of the left kidney was continued. A CRI of noradrenaline at 1 μg/kg/min maintained mean blood pressure at 50–73 mmHg. After releasing all the vascular clamps, the mean arterial blood pressure was 65 mmHg. The dog regained consciousness without any incidents.

**FIGURE 8:**
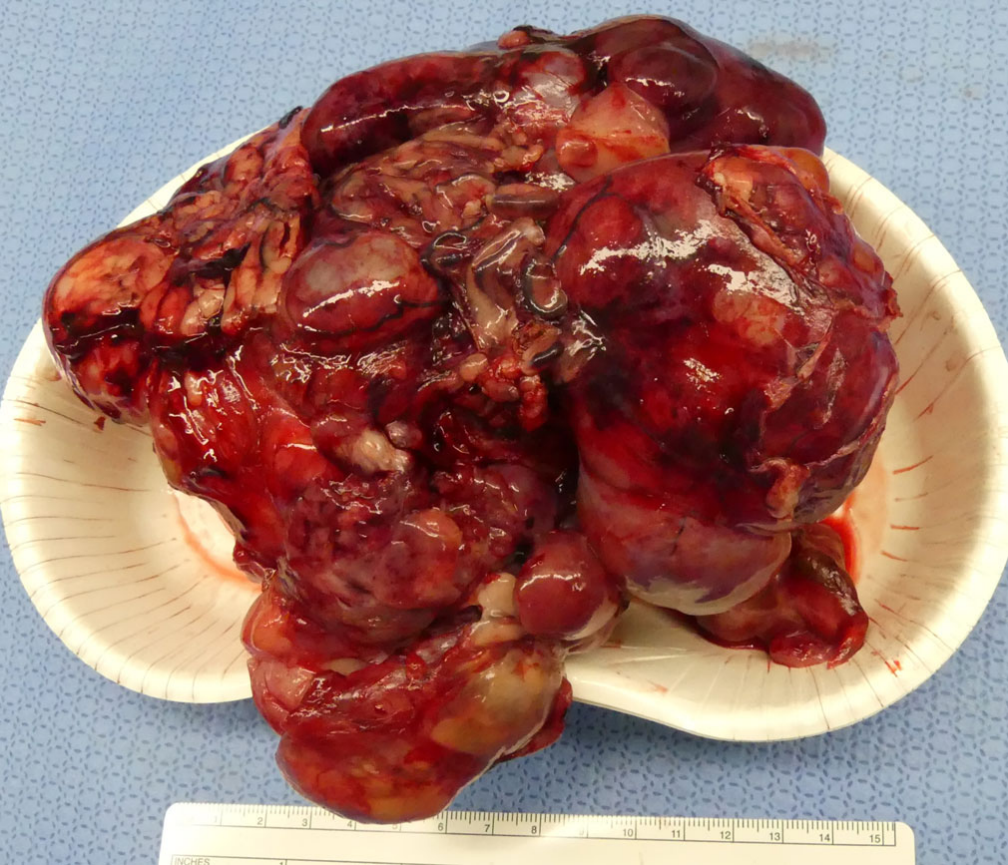
The resected mass involving the right kidney. The weight of the tumor was 953 g with a maximum diameter of 16.5 cm, yielding a weight‐to‐body ratio of 5.5%.

Histopathologic examination revealed that the extensive mass was RPS. The weight of the tumor was 953 g with a maximum diameter of 16.5 cm, yielding a weight‐to‐body ratio of 5.5% (Figure 8). Extensive bleeding and necrosis were evident inside the tumor. The tumor involved the right adrenal gland, and histopathologic examination confirmed that it invaded the adrenal tissue and infiltrated the perirenal fat. Furthermore, the mass in CVC was 8.4 cm in length, of unknown origin, and predominantly composed of connective tissue with minimally identified cellular components.

On postoperative day (POD) 1, the dog was generally stable. Blood test results, including renal parameters such as blood urea nitrogen (BUN) and serum creatinine (Cr), showed no elevations. Coagulation parameters exhibited only transient postoperative changes with no clinical significance. A summary of the laboratory findings is presented in Table 1. Postoperative blood pressure was supported with dopamine and dobutamine infusions at 2.5 μg/kg/min each until POD 1. Urine output was closely monitored during this period and remained stable at approximately 2–4 mL/kg/h, consistent with the volume of fluid administered. Continuous intravenous infusions of fentanyl and lidocaine were discontinued on POD 1. On POD 2, the dog remained stable without signs of pain‐related symptoms or respiratory compromise, and the thoracic drain was removed. Small amounts of food were introduced, and supportive IV fluid therapy was discontinued on POD 3. Appetite and overall condition gradually improved, and by POD 13, the dog had regained preoperative levels of food intake and activity. Hormone tests revealed a blood cortisol level of 0.7 μg/dL, while the post‐value in the ACTH stimulation test was 7.0 μg/dL. No abnormalities were observed in postoperative cortisol levels. The dog was discharged on POD 20. Blood tests revealed normal renal function. The complete blood test results are presented in Table 1.

After discharge, the dog was placed under the care of the referring hospital. No long‐term complications related to the surgery were observed. Blood test results, including BUN and Cr, remained within reference ranges, and the postoperative course was uneventful, with no clinical signs observed. At 1 year postoperatively, a follow‐up telephone inquiry with the referring hospital confirmed that the dog remained in good condition. Thoracic radiography and abdominal ultrasonography revealed no evidence of recurrence or metastasis.

## 4 DISCUSSION

In the present case, complete tumor resection required not only removal of the mass and associated caval thrombus but also prolonged and meticulous dissection from the CVC due to extensive infiltration into the surrounding tissues. Prolonged occlusion of the CVC to enable tumor dissection was anticipated preoperatively. In human medicine, clamping of the inferior vena cava during hepatic resection has been reported to result in decreased intraoperative superior vena cava pressure and systolic arterial pressure.^9^ During tumor dissection from the CVC, venous drainage from the left kidney was temporarily preserved by repositioning the tourniquet from the caudal to the cranial level of the left renal vein. However, continued occlusion of the CVC cranial to the left renal vein can increase venous pressure in the hindquarters, thereby impeding outflow from the left kidney and potentially resulting in renal dysfunction. In human patients, semi‐clamping of the infrahepatic inferior vena cava has been associated with postoperative acute renal failure.^8^ In dogs with adrenal tumors, en bloc resection of the ipsilateral kidney invaded by a tumor has been linked to an increased risk of short‐term mortality.^1^ In the present case, intraoperative measures were considered essential to mitigate the risk of renal failure following en bloc resection of the tumor, caval thrombus, and ipsilateral kidney. The use of VVB contributed to the maintenance of hemodynamic stability during prolonged CVC occlusion, thereby supporting preservation of renal perfusion. Based on these findings, intraoperative circulatory support with VVB may be recommended during CVC occlusion in similar cases to maintain perfusion of the contralateral kidney and to help preserve residual renal function. However, the use of VVB is technically demanding and associated with increased surgical costs. VVB may not be indicated in all cases requiring temporary caval occlusion. Further investigations are warranted to establish appropriate criteria for its application.

In the present case, although the CVC was extensively involved, CT did not reveal the presence of collateral circulation between the CVC and the azygos vein. A previous experimental study demonstrated that gradual occlusion of the suprarenal CVC could promote the development of collateral pathways to the azygos vein with the prevention of venous congestion‐related complications such as hindlimb edema or ascites.^16^ When such collateralization is present, en bloc resection of hepatic^17^ or adrenal tumors^18^, ^19^ involving the CVC may be accomplished without the need for VVB. However, the absence of collateral circulation in this case suggested that safe preservation of the CVC during tumor resection would not be feasible without the use of VVB. Moreover, the tumor exhibited marked infiltration into the surrounding tissues, and preoperative imaging indicated that dissection from the CVC would be time‐consuming. Although cavotomy at the level of the right phrenicoabdominal vein was considered technically feasible for thrombus removal, the procedure ultimately required 44 min and 43 s of temporary CVC occlusion. While the maximum safe duration of caval occlusion has not been definitively established, prolonged interruption of venous return between the liver and kidneys may pose a substantial risk of renal impairment. This case may highlight the clinical utility of VVB in enabling safe and effective resection of an extensively infiltrative adrenal tumor with associated caval thrombus.

RPS is rarely documented in dogs, with only a few reports available in the literature, indicating its rarity.^7^ RPS is characterized by local invasiveness and a high rate of distant metastasis, with tumor‐related mortality reported to range from 70% to 92.9%. The median survival time of affected dogs has been reported to range from 37.5 to 168 days.^7^ In our case, complete surgical resection of RPS was associated with long‐term survival. To achieve this, meticulous preoperative planning, including consideration of VVB based on CT imaging, is essential. Moreover, no previous studies have reported canine RPS cases involving the adrenal glands.^7^ In the present case, the sarcoma appeared to originate from the adrenal gland and had grown extensively, contributing to the development of RPS. The lesions observed differed from those previously reported, and the precise origin of the sarcoma remains unclear.

The Anthron bypass tube used in the present case has an outer diameter of 4 mm, making it suitable for use in dogs with jugular and femoral veins exceeding this diameter. Accordingly, its application is limited in smaller dogs where the vessel caliber is insufficient to accommodate the device. Preoperative assessment of the venous diameter using imaging modalities such as ultrasonography and/or CT may therefore be required to determine suitability. The tube is composed of a heparinized hydrophilic material that continuously releases a small amount of heparin for up to 16 h, thereby reducing the risk of intraluminal microthrombus formation.^20^ This duration appears sufficient to permit tumor dissection and CVC reconstruction under temporary bypass. However, the Anthron bypass tube was originally designed for use in human medicine to establish a bypass between the portal and femoral veins during total pancreatectomy,^10^ or between the portal vein and inferior vena cava during liver transplantation.^11^ As such, it is only commercially available in a standard length of 60 cm. In our case, two tubes were connected to achieve the necessary length to bridge the femoral and jugular veins, resulting in increased surgical costs. Therefore, the development of bypass tubes with smaller diameters and longer lengths, specifically adapted to the anatomical needs of small animal patients is desirable.

As for perioperative management, prednisolone was administered as a premedication in our case. At our institution, this agent is routinely used not only to prevent transient cortisol suppression associated with unilateral adrenalectomy but also to reduce the risk of hemorrhagic shock and postoperative hypoglycemia in extensive surgeries. However, the appropriateness of this administration remains debatable. During surgery, positive inotropic agents, including dobutamine, norepinephrine, and phenylephrine, were administered to maintain hemodynamic stability in response to anticipated blood loss and the use of VVB. Carperitide was administered for the preservation of renal function. It has been shown to exert renoprotective effects in experimental ischemic models of rats^21^ and dogs,^22^ and in a clinical study involving human patients undergoing abdominal aortic aneurysm repair.^23^ Nonetheless, the evidence supporting its efficacy remains limited. Nafamostat mesylate was also used intra‐ and postoperatively to prevent pancreatitis, which may be induced by manipulation and desiccation of the pancreas during extensive abdominal surgery. While its protective effects have been demonstrated in humans with post‐endoscopic retrograde cholangiopancreatography pancreatitis,^24^ its role in dogs with pancreatitis related with surgery has not been demonstrated. Further studies are needed to evaluate the efficacy and optimal application of these agents in similar clinical settings.

## 5 CONCLUSION

In conclusion, this is the first report of a clinical application of VVB during temporary caval occlusion to achieve complete en bloc resection of an extensive adrenal RPS with ipsilateral kidney and caval thrombus, followed by repair of a CVC incision. VVB may be a valuable technique for maintaining hemodynamic stability and reducing hemorrhage during complex oncological surgeries requiring temporary caval occlusion.

## AUTHOR CONTRIBUTIONS

All authors contributed to the study design and planning. Heishima T, DVM, PhD, Ishigaki K, DVM, PhD, Iida K, DVM, Takeuchi R, DVM and Asano K, DVM, PhD, Charter DJCVS: Were responsible for the surgery and perioperative management. Heishima T, DVM, PhD, Ishigaki K, DVM, PhD, Ueda T, DVM and Asano K, DVM, PhD, Charter DJCVS: Conducted the preoperative management and follow‐up evaluations. Kagawa Y, DVM, PhD, JCVP, ACVP: Performed the histopathologic assessment. All authors contributed to the writing of the manuscript and approved the final version.

## CONFLICT OF INTEREST STATEMENT

The authors declare no conflicts of interest.

## Data Availability

All data supporting the conclusions of this article are included within the article.
